# The Ultrasonographic Features of Shoulder Pain Patients in a Tertiary Hospital in South China

**DOI:** 10.1155/2020/3024793

**Published:** 2020-08-25

**Authors:** Li Jiang, Juanjuan He, Carl P. C. Chen, Dongfeng Xie, Yiying Mai, Boyu Yue, Zulin Dou

**Affiliations:** ^1^Department of Rehabilitation Medicine, The Third Affiliated Hospital, Sun Yat-Sen University, Guangzhou, China; ^2^Department of Neurology, The First Affiliated Hospital of Sun Yat-Sen University, China; ^3^Department of Physical Medicine & Rehabilitation, Chang Gung Memorial Hospital at Linkou and College of Medicine, Chang Gung University, Guishan District, Taoyuan City, Taiwan

## Abstract

**Methods:**

Patients with shoulder pain were recruited in an outpatient rehabilitation clinic at the Third Affiliated Hospital of Sun Yat-Sen University from January 1, 2017, to June 30, 2018. These shoulder pain patients with or without limitation in joint movement can be included in the study. All of them received musculoskeletal ultrasound scanning. Demographic and imaging data including age, gender, duration of shoulder pain, pain side, and pathologies found by musculoskeletal ultrasound imaging were collected and analyzed. Patients were divided into three groups: <45 years (young group), between 45 and 60 years (middle-aged group), and >60 years (elderly group). The rates of various shoulder pathologies were evaluated and compared between the groups.

**Results:**

This study recruited a total of 346 patients with shoulder pain. There were more female (62.1%) than male patients (37.9%), with the largest number of patients in the 45-60 years of age group (40.5%). Forty-eight percent of patients had shoulder pain within a period of 3 months. A total of 380 shoulders were assessed using musculoskeletal ultrasound imaging. The occurrence rate of subacromial disorder (83.8%) was the highest. The rate of supraspinatus tendinopathy, acromioclavicular joint degeneration, and adhesive shoulder capsulitis varied significantly between age groups (*P* < 0.05). The rate of acromioclavicular joint degeneration was the highest in the elderly group followed by the middle-aged and young groups (*P* < 0.0167). The rate of supraspinatus tendinopathy and adhesive capsulitis in the middle-aged and elderly groups was significantly higher than that in the young group (*P* < 0.0167).

**Conclusions:**

Musculoskeletal ultrasound can be a useful imaging tool in making an accurate diagnosis of shoulder pain. The occurrence rates of different shoulder pain pathologies in all age groups were thoroughly calculated in this study. More female and more subcoracoid disorder patients than western countries are attributed to repetitive lifting in daily life and work in this study. Correlations between these pathologies and their associated images can be a solid foundation for the development of artificial intelligence in diagnosing the cause of shoulder pain.

## 1. Introduction

Shoulder pain is a common complaint in rehabilitation and orthopedic outpatient clinics, with a prevalence rate between 2.4% and 26% [1]. In general, the prevalence of shoulder pain increases with age. Recurrent and prolonged shoulder pain contributes to unfavorable prognosis and decreased quality of life. Many pathologies can result in shoulder pain, such as rotator cuff tendinopathy, subacromial impingement, subacromial bursitis, and tendinitis of the long head of the biceps (LHB). Accurate diagnosis is crucial in making a treatment plan for patients with shoulder pain as wrong treatment strategy may lead to poor prognosis [2]. However, the distribution on the cause of shoulder 1pain remains unexplored in mainland China. The application of musculoskeletal ultrasound in diagnosing shoulder pain pathologies is still not widely practiced in China.

In the past, the diagnosis of shoulder pain is usually made according to medical history, clinical symptoms, and physical examination. However, uncertainty and wrong diagnosis may result [3]. As a result, further imaging evaluations, such as X-ray, magnetic resonance imaging (MRI), or musculoskeletal ultrasound, may be beneficial in making the correct shoulder pain diagnosis. MRI is radiation free but the disadvantages of this examination are that it is expensive and does not offer real-time images [4]. Studies have shown that musculoskeletal ultrasound imaging has good sensitivity and specificity in diagnosing shoulder pathology [5, 6]. Compared with MRI, musculoskeletal ultrasound imaging provides further advantages such as portability and cost-effectiveness and offers real-time images, which are helpful in observing small and occult lesions [4]. Therefore, musculoskeletal ultrasound has been widely used in the diagnosis of shoulder pain in developed countries. During the past several years, the outpatient clinic in the department of rehabilitation at the Third Affiliated Hospital of Sun Yat-Sen University in China has used the technology of musculoskeletal ultrasound imaging in examining hundreds of patients with shoulder pain and has collected thousands of relevant clinical imaging data.

The major aim of this study was to observe the epidemiological pattern on the causes of shoulder pain in China. The occurrence rates of the different diagnoses of shoulder pain based on ultrasound images were calculated in all age groups. We hypothesize that the crucial information can be used as robust evidences in the future development of ultrasound diagnostic artificial intelligence that can be used in imaging interpretation of shoulder pain pathologies.

## 2. Methods

This study was conducted from January 1^st^ of 2017 to June 30^th^ of 2018. A total of 346 patients with shoulder pain was recruited. Informed consent was assigned before participating in this study. The two main inclusion criteria were (i) shoulder pain with or without a joint range of motion limitation and (ii) patients were able to cooperate with the shoulder ultrasound examination apparatus. Patients with shoulder pain caused by spinal disease, shoulder joint fracture, trauma, tumor, systemic diseases, or post shoulder surgery were excluded from this study. Patients were divided into three age groups: the young group (<45 years), the middle-aged group (45-60 years), and the elderly group (>60 years). In addition, 12 healthy volunteers, 4 in each age group, were also recruited as controls and assessed by musculoskeletal ultrasound imaging.

### 2.1. Musculoskeletal Ultrasound Scanning

Patients were examined using the S-Nerve ultrasound system (Sonosite, USA). The 6–13 MHz linear transducer was applied. Two physiatrists with at least 3 years of experience in ultrasound diagnosis and image interpretation performed all the examinations. They also received one week of ultrasound operating training together to reduce experimental bias. During the examination, when both physiatrists were confronted with controversial images, they discussed meticulously together to make the correct diagnosis. Musculoskeletal ultrasound technical guidelines were applied to examine the following physiological soft tissue structures [7]: tendon of the long head of the biceps (LHB), subscapularis (Sub) tendon, subcoracoid (SC) bursa, acromioclavicular (A-C) joint, subacromial (SA) impingement, subacromial (SA) bursa, supraspinatus (SupraS) tendon, infraspinatus and teres minor (InfraS and Tm) tendons, and glenohumeral (G-H) joint.

Each patient was asked to sit on the revolving chair facing the physiatrist. When examining the LHB tendon, each patient was asked to place the arm in slightly internal rotation with the elbow flexed 90° and with palm up. The tendon of LHB between the greater and lesser tuberosities and its intra-articular course were examined, using the short- and long-axis planes. During the examination of the Sub tendon, each patient rotated the arm externally fixing the elbow joint on the iliac crest with the elbow flexed 90° and also with palm up. The short- and long-axis planes of the Sub tendon and its insertion on the lesser tuberosity were examined.

When checking the A-C joint, the transducer probe was placed in the coronal plane over the joint that is formed by the acromion and clavicle. During the examination of the SupraS tendon, each patient's arm was placed in the modified Crass position [7]. The SupraS tendon and subacromial bursa were evaluated along the long and short axes. Dynamic assessment of SA impingement was done by placing the transducer in the coronal plane with its medial margin at the lateral margin of the acromion. Then, shoulder abduction was done, and the sliding motion of the supraspinatus tendon was examined. SupraS and the bursa can be viewed passing deep to the coracoacromial arch. The posterior aspect of the shoulder was examined with the transducer placed over the posterior aspect of the G-H joint. InfraS and Tm tendons were examined as individual structures. The posterior aspects of the tendons of the G-H joint, posterior fossa, and glenoid labrum on their long axes during external and internal rotation of the arm were also observed.

### 2.2. Ultrasound Imaging Sign of Shoulder Pathologies

According to the literature [2], the signs of shoulder pathology in musculoskeletal ultrasound imaging can be summarized as follows.

LHB tendinitis: thickened tendons and effusions in tendon sheaths, obvious pain when pressure was applied locally. Tendon of LHB dislocation: absence of biceps tendon in the bicipital groove. Bursitis, including SC bursitis and SA bursitis: focal or diffuse bursal thickening of more than 2 mm transverse thickness with associated hypoechogenicity with or without bursal fluid. Rotator cuff tendinopathy: may be accompanied with thickened tendons and with heteroechoic findings. Rotator cuff calcific tendinitis was diagnosed in the presence of hyperechoic calcific deposition within the tendon. Partial-thickness tear of SupraS: partial tear of the tendon fibers occurs either at the bursal or at the articular surface that appeared as a focal hypoechoic or anechoic defect not traversing the entire tendon. Full-thickness tear: discontinuity of the tendon fibers resulting in communication between the articular and bursal surfaces appearing as a hypoechoic or anechoic defect. Subacromial impingement: pooling of the SA bursal content just lateral to the acromion during dynamic shoulder abduction evaluation.

A-C joint degeneration: ultrasound image showing evidence of A-C joint hyperosteogeny, rough cortex of the joint surface, and with or without joint effusion or capsular thickening. Adhesive capsulitis: decreased range of motion of G-H joint, and the possibility of glenohumeral effusion, and LHB tendon sheath effusion. In the present study, rotator cuff tendinopathy, rotator cuff calcific tendinitis, rotator cuff partial- or full-thickness tear, and subacromial bursitis were all regarded as subacromial disorders as suggested in the literature [3].

### 2.3. Data Analysis

Clinical data of shoulder pain patients were collected, which included age, gender, duration of shoulder pain, painful side, and musculoskeletal ultrasound imaging of the affected shoulders. Data analyses were performed using the SPSS 17.0 software package. Data were described as mean and standard deviation. The rate of occurrence comparisons among the three groups (the young group, the middle-aged group, and the elderly group) was performed using the chi-square test. The statistical significance was set as *P* < 0.05.

## 3. Results

A total of 346 patients with shoulder pain were recruited in this study. The age ranged from 18 to 90 years. There were more female patients than male patients. The shortest duration of shoulder pain was only 6 days, and the longest duration was more than 10 years. The ratio of patients with a joint range of motion limitation was higher (80.2%) than without motion limitation (19.8%). Epidemiologic characteristics of patients with shoulder pain are summarized in Table 1.

### 3.1. Rate of Occurrence of Various Shoulder Pathologies

**Figure 1:**
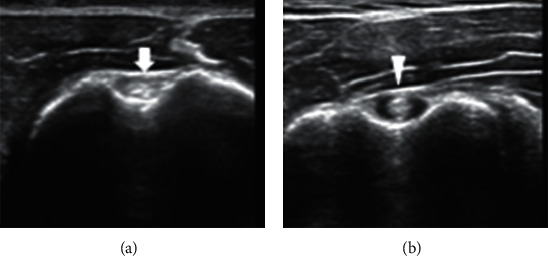
Ultrasound images of LHB tendon in healthy (a) and painful (b) shoulders. LHB tendon in healthy and nonpainful shoulder (arrow). LHB tendon in painful shoulder (arrowhead). The tendon was rounded and enlarged, with effusion in the tendon sheath.

**Figure 2:**
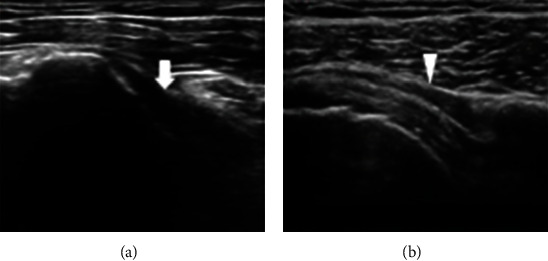
Ultrasound images of SC bursa in healthy (a) and bursitis in painful (b) shoulders. Subcoracoid bursa in healthy shoulder (arrow). In subcoracoid bursitis, thickened bursa with effusion can be observed (arrowhead).

**Figure 3:**
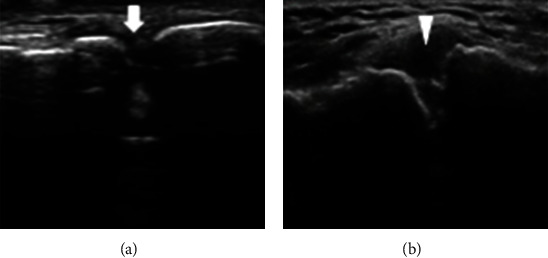
Ultrasound images of A-C joint in healthy (a) and painful (b) shoulders. A-C joint in healthy shoulder (arrow). In A-C joint degeneration and sprain, joint hyperosteogeny and capsular swelling can be observed (arrowhead).

**Figure 4:**
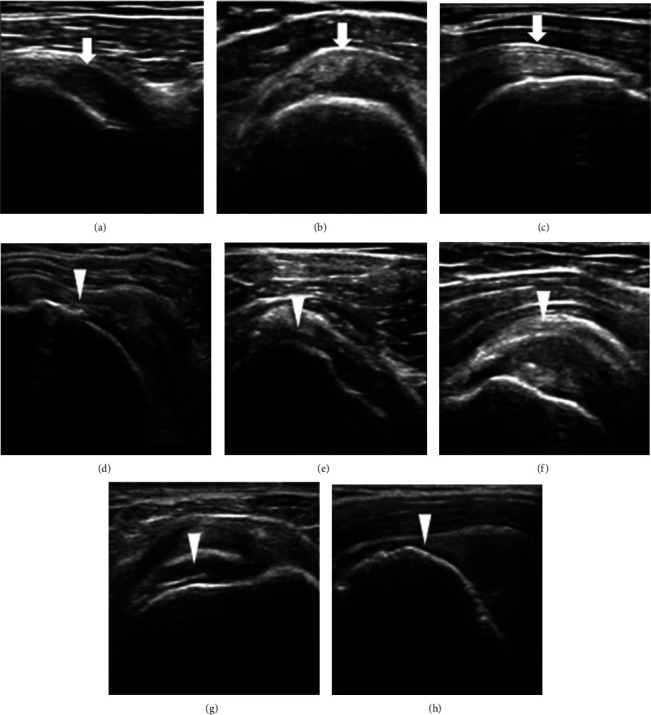
Ultrasound images of the subacromial area in healthy and painful shoulders. Healthy nonpainful shoulders (arrows): Sub tendon (a), SA bursa (b), and SupraS tendon (c). Painful subacromial disorders (arrowheads): Sub tendinopathy (d): inhomogeneous echogenicity and local hyperechoic finding within the tendon; SA bursitis (e): increased thickness of the bursa; SupraS calcific tendinitis (f): an obvious hyperechoic line within the tendon signifying calcification; partial thickness of SupraS tendon tears (g): dimpling of the deltoid muscle into the SupraS tear site; full-thickness tear of SupraS tendon (h): discontinuity of SupraS tendon fibers, and increased hypoechoic signals at the tear site.

A total of 380 shoulders were assessed using musculoskeletal ultrasound imaging. The most common pathology was due to subacromial disorders (83.8%), including rotator cuff tendinopathy (48.5%), calcific tendinitis (16.3%), partial- or full-thickness tear (15.5%), and subacromial bursitis (59.5%) (Table 2). Ultrasound images of these images are shown in Figures 1–4.

### 3.2. Comparisons of the Rate of Occurrence of Various Shoulder Pathologies in Different Age Groups

The percentages of shoulder pain in the young group was 23.4% (*n* = 89), in the middle-aged group was 41.6% (*n* = 158), and in the elderly group was 35% (*n* = 133). The rate of occurrence in terms of percentages on common shoulder pathologies between the 3 groups was compared (Table 3).

## 4. Discussion

In this study, we analyzed the epidemiology and imaging characteristics of shoulder pain in a tertiary hospital using musculoskeletal ultrasound. To our knowledge, this is the first epidemiological and imaging characteristic study on outpatients with shoulder pain in mainland China using ultrasound. Some epidemiological studies have shown that the prevalence of shoulder pain in women is higher than that in men [8]. In this study, there were more female patients than 1male patients, which was consistent with these previous epidemiological studies. In addition, it was reported that the rate of shoulder pain among people aged 45 to 64 years was the highest [8]. In this study, shoulder pain was most common in the 45-60 years of age group and with an average age of 53.5 years. Currently, the relationship between dominant hand and shoulder pain is controversial. In this study, patients with left shoulder pain are roughly equal to that of right shoulder pain and have no relationship to the side of the dominant hand. In the study of Ostor et al., the median duration of shoulder pain was 10 weeks, with patients mainly from primary care units [9]. In another study conducted by Juel and Natvig, the average duration was 26 weeks, with patients coming from secondary care units [10]. Most patients in this study had shoulder pain duration within 3 months. The duration of shoulder pain in this study can be significantly shorter; this is due to the fact that patients have easier access to the tertiary hospital medical care without having to visit a general practitioner first in China.

Due to the different diagnostic criteria, the rate of shoulder pathology reported in different studies varies greatly [8, 10, 11]. In the study by Murthi et al., the prevalence of pathological changes in the LHB tendon is 40% [12]. However, Patton suggested that primary tendinitis of the LHB accounts for only 5% of tendon pathology. Redondo-Alonso et al. suggested that the occurrence of secondary tendinitis of LHB is low and is usually associated with other shoulder disorders such as supraspinatus tendinopathy, subacromial impingement, and glenohumeral arthritis [13]. In this study, the rate of LHB tendinitis was 23.2%, which was lower as compared with the study by Murthi et al. A possible explanation is that all the patients in this study were assessed by musculoskeletal ultrasound imaging to establish an accurate diagnosis, not using physical examination. Also, in this study, the LHB lesion was diagnosed as an individual pathology and was not distinguished as primary or secondary tendinitis.

The subcoracoid bursa is situated below and medially to the acromion near the joint capsule and the subscapularis tendon. The function of the bursa is to minimize the friction between the coracoid process and the subscapularis muscle [14]. Pathological lesions within the subcoracoid bursa have rarely been reported. In this study, we found a considerable number of patients with subcoracoid bursitis using ultrasound imaging. Repetitive lifting in life and work styles can be a cause of a higher occurrence rate of subcoracoid bursitis in China. This is the first study that has reported that subcoracoid bursitis should be ruled in as one of the possible causes of shoulder pain. It was reported that the rate of A-C joint degeneration is 48%-82% in the MRI images of asymptomatic patients [15]. In this study, the rate of A-C joint degeneration in causing shoulder pain was 40.5%. The incidence of A-C joint degeneration increases with age [11]. In this study, the rate of A-C joint degeneration in the elderly group was the highest, followed by the middle-aged group and the young group, which was consistent with the findings done in other studies [16].

Subacromial disorders in different literature involve rotator cuff tendinopathy (tendinitis), calcified tendinitis, partial- or full-thickness tear, and subacromial bursitis. Ottenheijm et al. considered that subacromial disorders were the most common disorders affecting the shoulder [3]. The rate of subacromial disorders reported was from 36% to 86% [9]. This study showed that the rate of occurrence of subacromial disorder was 83.8%. Rotator cuff consists of subscapularis, supraspinatus, infraspinatus, and teres minor tendons. As we know, tendinopathy is an age-related degenerative overuse pathology. In Tran et al.'s study, the rate of rotator cuff tendinopathy was 36% [2]. In this study, the rate of occurrence of rotator cuff tendinopathy was 48.5%. The possible cause of this rate difference is that in Tran et al.'s study, shoulder pain patients came from community referrals. In other words, patients with shoulder pain may not even be referred to tertiary hospitals at all. But in this study, patients with shoulder pain came directly to our outpatient clinic for treatment, possibly contributing to a higher percentage of rotator cuff tendinopathy. Furthermore, it was reported that the incidence of rotator tendinopathy increases with age. In the study by Leong et al., patients above 50 years of age are associated with an increased risk of rotator cuff tendinopathy [17]. In this study, we found that the occurrence rates of supraspinatus tendinopathy in the middle-aged and elderly groups were higher than the young group, which is in accordance with the literature written by Leong et al. [17].

Studies have reported that the prevalence of calcific tendinitis in the rotator cuff is between 6.8% and 54% in patients with shoulder pain [18]. Eighty percent occurs in the supraspinatus tendon [19]. In this study, we found that calcific tendinitis was observed in 16.3% of the patients with shoulder pain and occurred mainly in the supraspinatus tendon. Rotator cuff tear mainly occurs in the supraspinatus tendon and rarely occurs in the subscapular and infraspinatus tendons [20]. The occurrence rates of rotator cuff tear range from 5% to 39%, and musculoskeletal ultrasound has good sensitivity and specificity in the diagnosis of rotator cuff tear [3, 20]. In this study, the rate of supraspinatus tendon tear was observed to be 15.5%, which was within the range of the reported data.

Musculoskeletal ultrasound is an accurate imaging tool in the diagnosis of subacromial bursitis [21]. In the study conducted by Tran et al., the occurrence rate of subacromial bursitis is 68% in shoulder pain patients [2]. In this study, the occurrence rate of subacromial bursitis was 59.5%, which was significantly lower than the percentage reported by Tran et al. A possible explanation is that subacromial bursitis is caused by repetitive overhead activities [22]. Similar to subcoracoid bursitis, lifestyle differences may be the cause of this rate difference in our study. Studies have reported that the occurrence rate of subacromial impingement in shoulder pain patients is approximately 35-65% [23]. However, the rate of subacromial impingement in the present study was only 28.7%. This may be due to the fact that subacromial impingement was diagnosed in other studies using the physical examination method [3]. The painful sensation during shoulder manipulation may be misinterpretedas subacromial impingement. But in our study, subacromial impingement was accurately diagnosed under ultrasound dynamic real-time testing. The sliding action of the supraspinatus tendon into the shoulder joint provided the golden standard in diagnosing subacromial impingement. As a result, false positive diagnosis of subacromial bursitis using the physical examination method can be avoided.

In the past, the diagnosis of adhesive shoulder capsulitis was based on clinical symptoms and physical examinations. Presently, literature has suggested that ultrasound imaging is the preferred imaging modality for the diagnosis of adhesive shoulder capsulitis [24]. The occurrence rate of adhesive shoulder capsulitis can be up to 55% in patients with shoulder pain [9, 25]. In this study, the occurrence rate of adhesive capsulitis was 11.8%, mainly affecting the middle-aged and elderly groups. Again, through the dynamic real-time evaluation of the shoulder joint, the diagnosis of adhesive capsulitis can be made more precisely, explaining why the occurrence rate is lower as compared with other studies [26].

### 4.1. Limitations

There are some limitations in this study. This study was conducted in one tertiary medical center only. In future related studies, shoulder pain patients from multicenters will be recruited as well. In China, patients with shoulder pain can visit any department freely. For instance, patients with severe shoulder pain may visit orthopedic outpatient clinic first and receive surgical treatments subsequently. These patients may not have the opportunity to receive ultrasound imaging evaluation first.

As a result, further collaboration with the orthopedic department is needed in order for the shoulder pain epidemiological data to be more comprehensive. Additionally, we did not do a reliability test before enrollment of the participants in this study.

## 5. Conclusion

This study thoroughly explored the epidemiology and the ultrasonographic features of shoulder pain among all age groups in south China. Accurate shoulder pain diagnoses were made from musculoskeletal ultrasound images. This study has discovered some significant findings such as that the occurrence rate of subcoracoid bursitis was much higher as compared with other studies. The occurrence rates of subacromial bursitis and adhesive capsulitis were actually lower as compared with other literature. Since hundreds of patients were recruited, correlations between the diagnoses of shoulder pain and thousands of musculoskeletal ultrasound images can be made. These correlations can be used as solid evidences in the development of artificial intelligence in ultrasound imaging interpretation of shoulder pain pathologies. In all, ultrasound is a reliable tool that can be used in achieving an accurate diagnosis of shoulder pain and should be widely used in outpatient clinical settings.

## Figures and Tables

**Table 1:** Epidemiologic characteristics of the recruited patients.

Characteristic		Mean (SD)/*N* (%)
Age (years)		53.5 (13.1)
Age group	<45 years	86 (24.9%)
	45-60 years	140 (40.5%)
	>60 years	120 (34.7%)
Gender	Female	215 (62.1%)
	Male	131 (37.9%)
Pain side	Left	140 (40.5%)
	Right	172 (49.7%)
	Bilateral	34 (9.8%)
Pain duration	<3 months	166 (48.0%)
	3 to 6 months	78 (22.5%)
	>6 months	102 (29.5%)

**Table 2:** Confirmed shoulder pain pathology using musculoskeletal ultrasound imaging.

Pathology		*N* (%)
Tendon of LHB pathologies	Tendinitis	88 (23.2%)
	Tendon dislocation	3 (0.8%)
SC bursitis		52 (13.7%)
Sub tendon pathologies	Tendinopathy	55 (14.5%)
	Calcific tendinitis	9 (2.4%)
A-C joint degeneration		154 (40.5%)
SA impingement		109 (28.7%)
SA bursitis		226 (59.5%)
SupraS tendon pathologies	Tendinopathy	96 (25.3%)
	Calcific tendinitis	53 (13.9%)
	Partial-thickness tear	45 (11.8%)
	Full-thickness tears	14 (3.7%)
InfraS and Tm tendon pathology	Tendinopathy	33 (8.7%)
Adhesive capsulitis		44 (11.6%)

Abbreviations: LHB tendon: long head of the biceps tendon; Sub: subscapularis muscle; SC: subcoracoid; SA: subacromial; A-C joint: acromioclavicular joint; SupraS: supraspinatus muscle; InfraS and Tm: infraspinatus and teres minor muscle.

**Table 3:** Comparison of shoulder pathologies in different age groups.

Pathology	Young group *N* (%)	Middle-aged group *N* (%)	Elderly group *N* (%)	*P*
LHB tendinitis	23 (25.8%)	31 (19.6%)	34 (25.6%)	0.386
SC bursitis	8 (9.0%)	22 (13.9%)	22 (16.5%)	0.274
Sub tendinopathy	11 (12.4%)	19 (12.0%)	25 (18.8%)	0.213
A-C joint	6 (8.1%)	67 (42.4%)^a^	81 (60.9%)^ab^	0.001
SA impingement	22 (24.7%)	54 (32.9%)	33 (24.8%)	0.136
SA bursitis	55 (61.8%)	97 (61.4%)	74 (55.6%)	0.535
SupraS tendinopathy	8 (9.0%)	42 (26.6%)^a^	46 (34.6%)^a^	0.001
SupraS calcific tendinitis	9 (10.1%)	20 (12.7%)	24 (18.0%)	0.205
SupraS tendon tear	10 (11.2%)	21 (13.3%)	28 (21.1%)	0.084
InfraS and Tm tendon pathology	7 (7.9%)	15 (9.5%)	11 (8.3%)	0.889
Adhesive capsulitis	3 (3.4%)	22 (13.9%)^a^	19 (14.3%)^a^	0.022

^a^ *P* < 0.0167 compared with the young group; ^b^*P* < 0.0167 compared with the middle-aged group. Abbreviations: LHB: long head of the biceps tendon; Sub: subscapularis muscle; SC: subcoracoid; SA: subacromial; A-C joint: acromioclavicular joint; SupraS: supraspinatus muscle; InfraS and Tm: infraspinatus and teres minor muscle.

## Data Availability

The data used to support the findings of this study are available from the corresponding author upon request.
